# New device for saphenous vein-to-aorta proximal anastomosis without side-clamping

**DOI:** 10.1186/1749-8090-2-22

**Published:** 2007-05-04

**Authors:** Ernesto Tappainer

**Affiliations:** 1 Cardiac Surgery Unit, "Carlo Poma" Hospital, Viale Albertoni 1, 46100 Mantua, Italy

## Abstract

**Background:**

Side clamping to perform saphenous vein-to-aorta proximal anastomosis is a well known cause of cerebral embolization during coronary bypass surgery. Automatic and manual devices have been introduced to avoid aortic clamping and facilitate proximal anastomosis but the manual ones only allow the traditional hand-sewing running suture. Nevertheless, they are not easy to use and very expensive to buy.

**Methods:**

We developed a simple object that helps to perform a manual proximal anastomosis without the need to clamp the side of the aorta. This device is a steel bar which blocks the aortic hole and simultaneously it provides a slit to receive the needle. Through the slit comes out a thin, sharp, straight, but also well directed and predictable jet of blood which could be easily controlled during the suture.

**Results:**

The function of the object is quite different from other devices. Nothing is deployed in the aorta. The object is only placed on the aorta with the small appendage slipped into the hole. The main advantage of the device is that while manipulation of the aorta is avoided no foreign bodies are incorporated in the suture and – most importantly – the aortic intima is not touched at all. The main drawback of the device is the blood jet coming from the slit so that the blood pressure has to be lowered by vasodilators during the anastomosis. Moreover, the suture has to change direction and the needle has to enter the aortic wall first to slip out through the slit.

**Conclusion:**

The object was named "Slit Device" and is not a routine instrument. It would be only an alternative to other anastomotic devices with the same surgical indications. In the case of ascending aortic disease and saphenous vein grafting, the Slit Device avoids aortic clamping thereby preventing atheroembolism and also avoiding the need for hypothermic circulatory arrest in patients with unclampable aorta.

## Background

**Figure 1:**
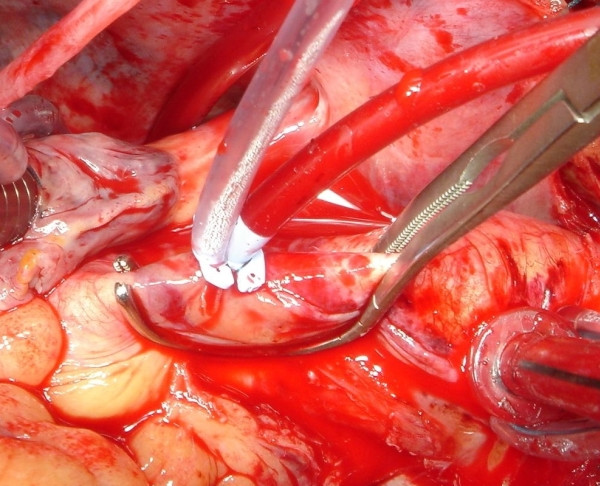
Example of side biting clamp for proximal anastomosis. The punch of 4.5 mm will be inserted in the hole of the aortic vent.

Coronary artery revascularization is the most important treatment of coronary artery disease. Coronary artery bypass grafting is the surgical way to accomplish this. Revascularization by arterial grafts – i.e. bilateral internal mammary arteries – is the gold standard surgical technique and it is performed more often today than in the past [1-3]. Nevertheless, for many reasons, saphenous vein grafting is still the norm and is a widespread technique in elderly patients or in emergency situations. Thus, even though total arterial revascularization is preferable, the patient population has become older and emergency situations are still frequent so that the need to use vein grafts remains high [3]. Vein grafts need a proximal anastomosis which is usually performed on the ascending aorta. Hand-sewing running suture is the most effective way to perform the anastomosis. It is easy, reproducible and inexpensive, but it requires a bloodless and stable field. In cases of vein-to-aorta proximal anastomosis it is obtained by a partial occluding, side-biting clamp on the aortic wall (Figure 1). Sometimes atherosclerosis makes it difficult with a high risk of atheroembolism. Side clamping to perform proximal anastomosis is a well known cause of cerebral embolization during coronary artery bypass grafting [4-7]. Moreover, the increasing age of patients increases the risk of embolization because of more extensive vascular alterations in older patients [3].

**Figure 2:**
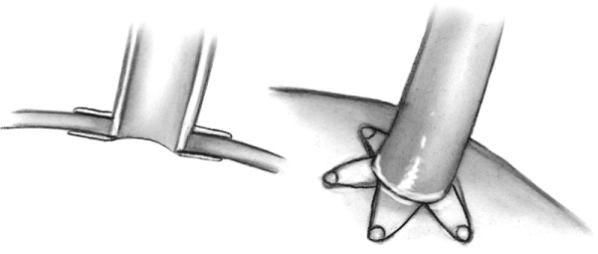
Drawing of anastomosis by an automatic device.

An improvement in the hand-sewing technique was the single cross clamping for both distal and proximal anastomoses [8], but it cannot be used in the off-pump technique. A combination of increasing age, pathological aorta and evolution of the off-pump technique stimulated the development of various distal and proximal anastomotic devices and now many of these have been introduced by manufacturers. These objects could be divided into two groups: automatic proximal anastomotic devices and manual proximal anastomotic devices (Table 1). Automatic devices release metal mechanisms which replace the hand-sewing running suture (Figure 2). For this reason they are not well accepted by surgeons, but they could be useful in critical situations. Moreover, there is the problem of a blood-exposed, non-intimal surface [9] and fear about long-term patency of the grafts [3]. Some devices require a dangerous manipulation of the vein graft and/or impose a 90 degree angulation between vein graft and aorta so that the graft cannot be placed in the desired direction. Manual devices allow a traditional hand-sewing running suture. They create a bloodless field around the aortic hole where anastomosis has to be performed. This is achieved by deploying concave membranes into the aorta. The membrane closes the hole from the inside but it allows the movements of the needle [10-12]. Nevertheless, these commercially available manual devices are somewhat difficult to use and very expensive to buy. Moreover, they have the potential to damage the aortic wall because they have to be in contact with the aortic intima – i.e. the most internal layer of the aortic wall. The cost of these devices is high and they are not easy to use. I have developed a simple, cheap device to perform a proximal anastomosis without the need of a clamp.

**Table 1:** 

**Automatic proximal anastomotic devices:**
Symmetry Aortic Connector System (St. Jude Medical, Minneapolis, MN, USA)
Cardica Pas-Port II anastomosis system (Cardica, Redwood City, CA, USA)
CorLink device (Bypass Ltd, Herzelia, Israel)
One-Shot Vascular Anastomotic Dev (Horologe Factory, Jinan City, Shandong, China)
Spyder Proximal Anastomotic Device (Medtronic, Minneapolis, MN, USA)

**Manual proximal anastomotic devices:**

Novare Enclose II (Novare Surgical Systems, Cupertino, CA, USA)
Heartstring Proximal Seal System (Guidant Corporation, Santa Clara, CA, USA)

## Methods

### History of the new object

Many years ago an unclampable aorta forced me to stop extracorporeal circulation for every aortic bite during the proximal anastomosis. At the same time, I had to cover the hole with a finger while the pump was resumed repeatedly during every bite to the vein. Immediately I looked for something to block or curtail the loss of blood.

**Figure 3:**
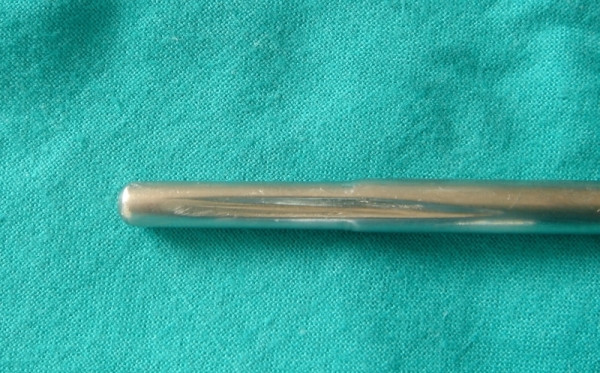
Object 1. It is a simple steel bar with a lateral furrow.

**Figure 4:**
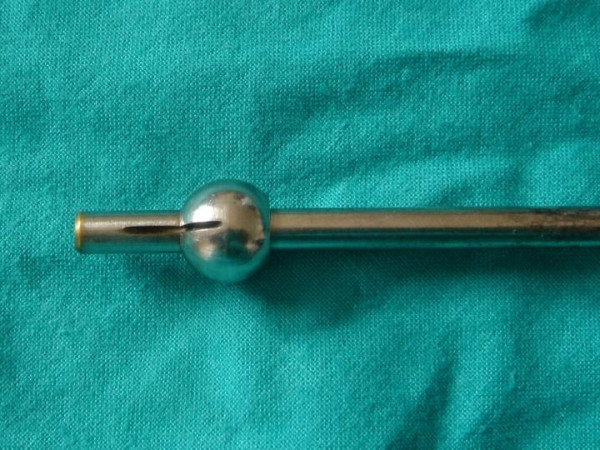
Object 2.

**Figure 5:**
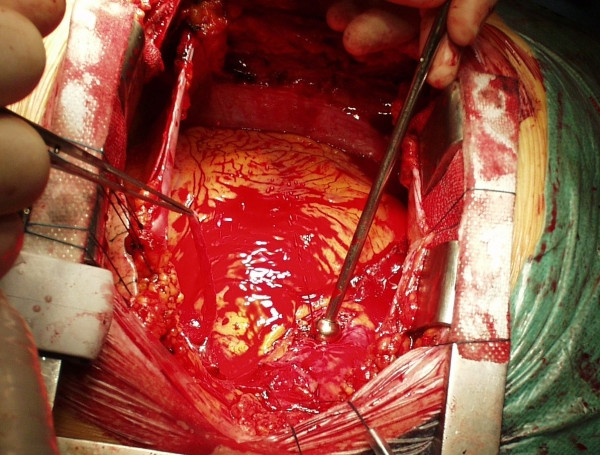
Object 2 during the test. The blood jet is very sharp and easy to control.

At first I prepared a steel bar of 5 mm in diameter for insertion in the aortic hole, with a lateral furrow to take the needle for the suture (object 1)(Figure 3). I thought that in critical cases of unclampable aorta rather than go to a hypothermic circulatory arrest otherwise required in these patients [11], I could try to perform the anastomosis even if a stream of blood flowed through the lateral furrow of the bar. After trying unsuccessfully to make some stitches, I realized that many problems needed to be solved. First the furrow was too large. It would have to become a narrow slit so that it could transform the stream of blood into a sharp jet. Second the bar should have a rest-point to understand where the end into the aorta was. Third, I realized that instead of doing the traditional suture where the needle enters the aortic hole first to pass the aortic wall from inside to outside, using a slit-object the needle has to enter the aortic wall first and to exit through the aortic hole and through the slit. Even if diseased, the aortic wall retains a minimum of elasticity, which allows the needle-holder to press the wall, push the tip of the needle against the bar into the furrow and rotate the needle just enough to drive the tip out. On the other hand, it would be ineffective to press the needle-holder against the steel bar. I also realized that it would be useful to use the 5-0, half-circle needle rather than the usual 6-0, 3/8 one. Then, I looked for a bar with a ball. I found it through the plumber in the hospital (object 2). A slit was made in the ball to receive the needle (Figure 4). However, it did not work because it was impossible to grasp the tip of the needle and so the idea was abandoned. Nevertheless, I learned that the blood-jet from the slit could be easily controlled if it was thin, clear and sharp (Figure 5).

When I used some of the devices introduced by the manufacturers to facilitate proximal anastomosis, I realized that the cost was very high and they were not easy to use. It seemed to me they were not easier to handle than an object with a sharp jet of blood. Therefore, I returned to my original idea. I thought that if I scooped out the ball I could probably grasp the tip of the needle.

**Figure 6:**
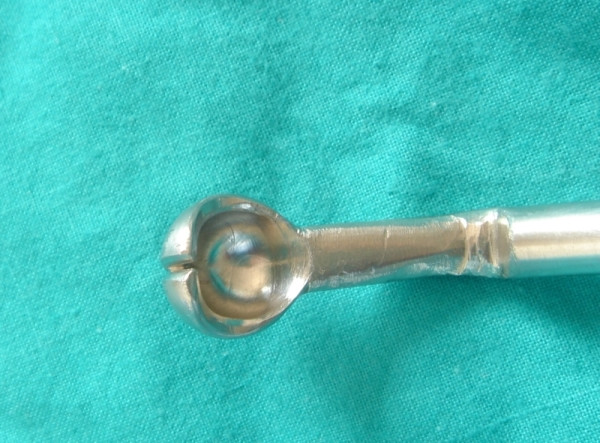
Object 3.

**Figure 7:**
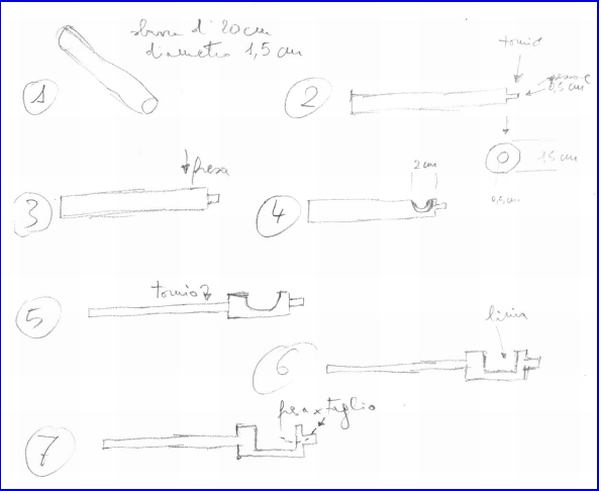
The original author's drawing for the mechanic.

**Figure 8:**
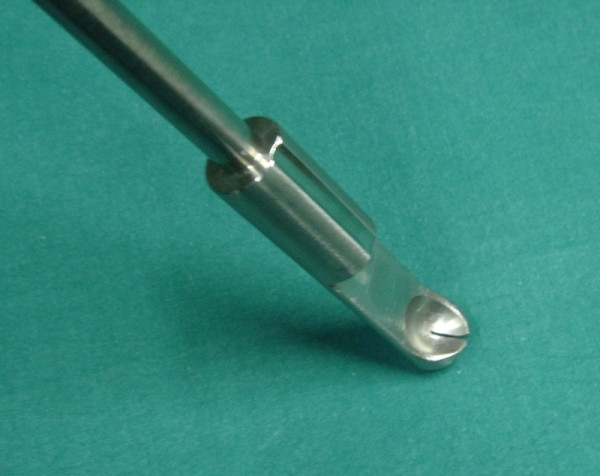
Object 4. It was too heavy and cumbersome.

**Figure 9:**
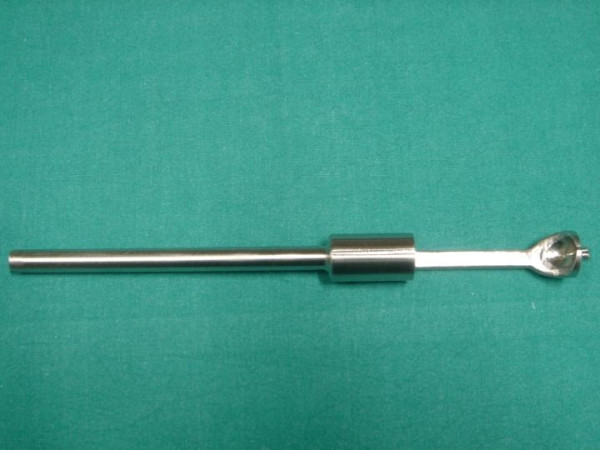
Object 5.

**Figure 10:**
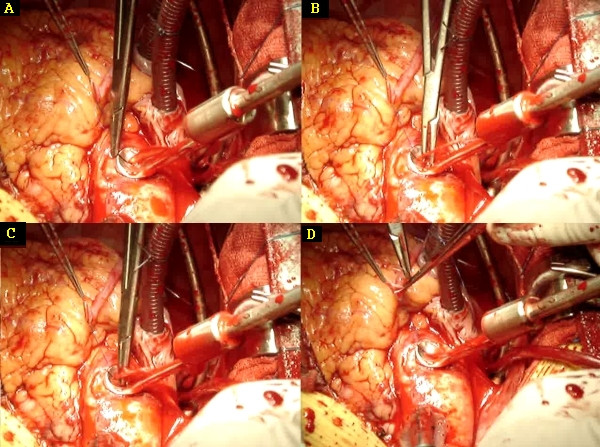
Object 5 during the first anastomosis. Low resolution frames of a videoclip. A: the needle enters the aorta first. B: the tip of the needle is sticking out. It cannot be seen, it has to be felt by the needle-holder. C: the tip of the needle is grasped by the needle-holder. D: the needle enters the vein graft.

**Figure 11:**
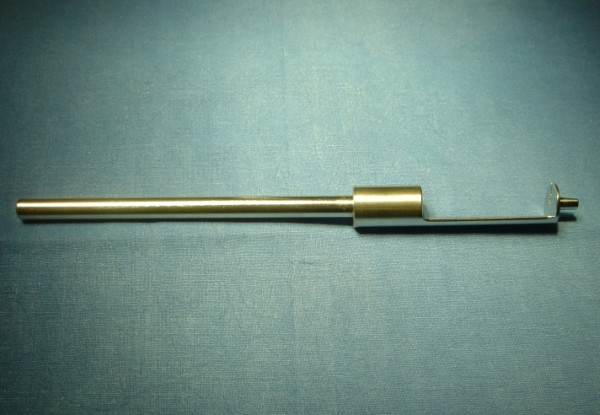
Object 6.

**Figure 12:**
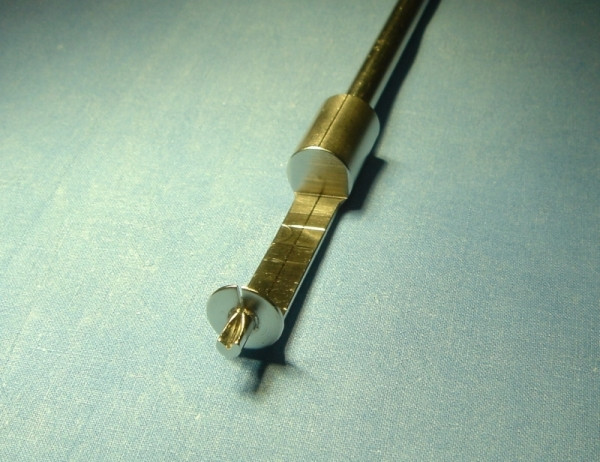
Object 6 again.

**Figure 13:**
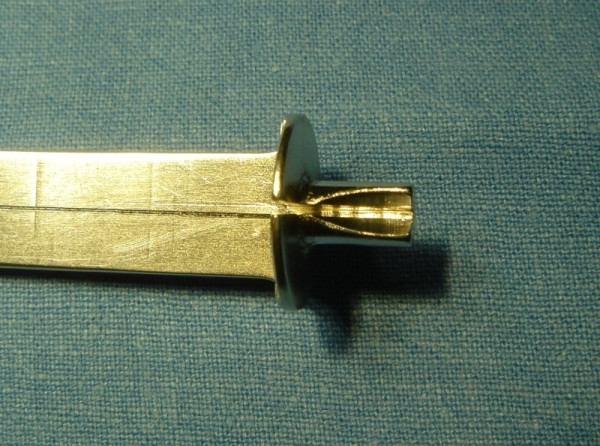
Object 6. The slit is still too large and it cannot be used in off-pump patients.

On 14^th ^January 2006 the Ethics Committee of Azienda Ospedaliera "C. Poma", Mantua, gave me written consent to develop the instrument for proximal anastomosis, which we named "Slit Device". At first I prepared a scooped out ball like a little spoon with a slit (object 3) (Figure 6). It did not work because it did not remain fixed over the hole. Even if it seems somewhat simple, I learned that the object must have a 5 mm appendage that enters the aortic hole, as the previous bar did. Moreover, it should have an enlargement to give an adequate weight so that it could be abandoned without being pushed out of the hole by the blood pressure. The same enlargement would be useful to stop the blood jet. After this, I incorporated all these reflections in a drawing from which the device has been developed by hand by an experienced mechanic (Figure 7). The next object (object 4) was too heavy and cumbersome (Figure 8). Later, another (object 5) was much better and was used eventually with good results for one vein-to-aorta anastomosis on a patient with a diseased wall of the aorta (Figure 9 – 10). After this test, I realized that the slit in the appendage had to be flared to drive the needle more easily. I also realized that the object did not need to look like a spoon, which was the remnant of the idea of the scooped-out ball. With a new simplified shape (object 6) so that a mechanism could be implemented in the future to reduce the blood jet, some other patients were satisfactorily treated (Figure 11, 12, 13). The latter were on-pump patients because the slit of the horizontal plate was too large, yet.

**Figure 14:**
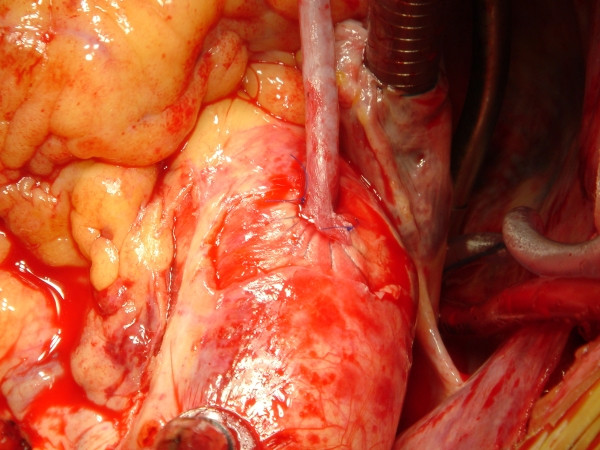
The first anastomosis. The object 5 was used. Anastomosis is not very graceful but it is effective.

Seven different objects were made. The first two objects were provided from the plumber of the hospital. The first was of simple steel and the second of brass. They were sterilized to try one stitch only but they were unusable. After the Ethic Committee's consent, all other objects for testing were made of stainless steel 316L. The object 5 was used to complete the first anastomosis (Figure 14). The object 6 has proven to be useful in selected cases. The last object, with the appendage curved a little, was carved out by a laser machine but has not been tested yet. However, the definitive device should be made of plastic so that the slit of the horizontal plate could be even more narrow. In this case the horizontal plate could be progressively thinner near the slit so that when doing the stitches the needle could force the slit and, at the same time, the blood jet would be reduced again. Some other simple mechanisms to control the blood through the slit are under study and patent.

## Results

### The Slit Device

**Figure 15:**
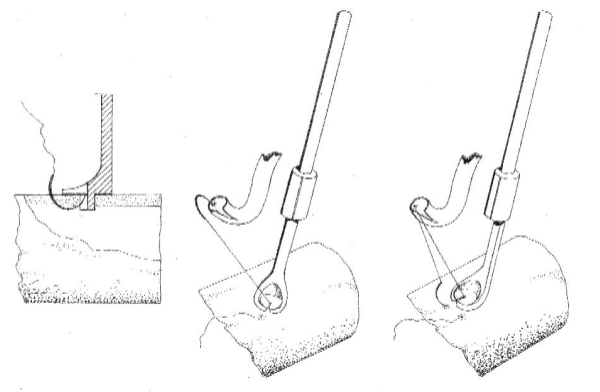
Object 5. Drawing of the technique for the suture.

**Figure 16:**
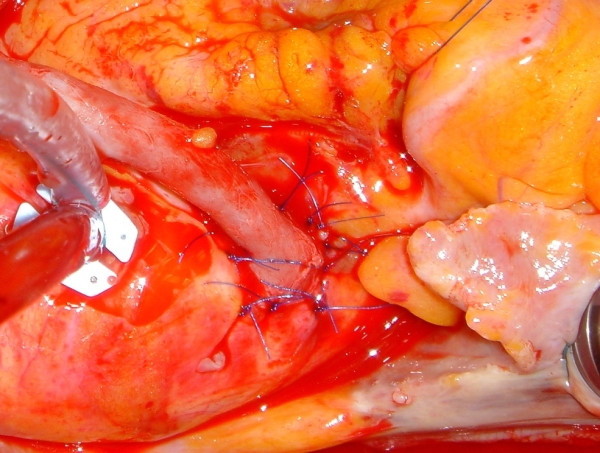
The anastomosis was made by interrupted suture. The device was employed for the first 4 opposite stitches in the aortic wall only. See text for explanation.

Slit Device is not a routine instrument. It is only an alternative to the other two manual anastomotic devices, with the same surgical indications: avoiding aortic clamping in the case of aortic disease. Similarily, when using them, some variations have to occur in surgical practice. First, the direction of the suture, i.e. the direction of the bites of the needle, is different in that the needle enters the aortic wall from the adventitia towards the intima. After that, the tip of the needle knocks against the flared appendage of the object, the flare drives it into the slit, then the needle is rotated by the needle-holder until the tip comes out through the slit of the device so that it could be grasped by the forceps or the needle-holder(Figure 15). A 5-0 half circle needle is needed, thus bigger than usual. This needle has a 90 cm long thread to allow distant suture (parachute technique). Near the end of the suture the device is removed, a finger stops the blood, the two arms of the thread are pulled, the vein is brought close, the coils of thread are pulled by a hook and finally the last stitch is whipped in. Alternatively, to lose as little blood as possible through the slit in off-pump patients, you could apply only four single separate stitches at the four cardinal points of the aortic hole using the device, then remove the device stopping the hole with your finger, pass the same four stitches in the vein graft, bring the vein graft closer removing the finger and pull the four separate threads by four thin, soft tourniquets so that the aortic hole is covered by the vein. Then whip the remaining stitches in as needed and tie them, release the four tourniquets and finally tie the first four stitches. In this way the anastomosis is performed by a less conventional interrupted suture instead of the usual running one (Figure 16).

Like the other one, Slit Device has many advantages and some problems. An important characteristic is that nothing is deployed into the aorta. The device is only placed on the aorta with the small appendage slipped into the hole. Its main advantage is that manipulation of the aorta is avoided, no foreign bodies are incorporated in the suture and, most importantly, the aortic intima is not touched at all. The advantages are listed in Table 2.

**Table 2:** 

Advantages	
Simplicity	no complex mechanisms easy training
Sturdiness	no breakable parts
Economy	it can be used for many anastomoses in the same patient if made of plastic it can be used for many patients if it is made of stainless steel
Versatility	it allows the possibility of deciding whether to perform distal or proximal anastomoses first it allows giving the right inclination to the graft.
Safety	nothing is deployed in the aorta no manipulation of the aorta no foreign bodies are incorporated in the suture no touching of the aortic intima

Even though there is no extensive clinical experience a disadvantage seems to be the need for manual dexterity for the rapid blind manoeuvres – scalpel, finger, punch, finger, device – which are the same with many other devices. Indeed, the main drawback of the device is the blood jet from the slit so that the blood pressure has to be lowered by vasodilators during the anastomosis. However, sudden jets of blood which are not sharp, not well directed, not predictable as with the Slit Device are frequent with other devices, too. Other disadvantages are listed in Table 3.

**Table 3:** 

**Disadvantages**	
Need for manual dexterity	
Need for rapid blind manoeuvre	scalpel, finger, punch, finger, device (but it is the same thing with the other devices)
Inversion of the suture	could be not acceptable to everyone
Need for a bigger needle	large enough to pass through the aorta and the device itself
Not a completely bloodless field	blood pressure has to be lowered by vasodilators

Thus, Slit Device belongs to manual anastomotic devices, such as Enclose II and Heartstring, but its function is completely different. It is not a sophisticated device like them. Nevertheless, like the others it could be safer than the partial occluding clamp in off-pump patients, too [13]. Slit Device can be useful in patients with severe atherosclerotic disease of the ascending aorta where a clamp cannot be applied.

## Conclusion

Complete arterial revascularization and hand-sewing suture are the most effective procedures in coronary artery bypass surgery. Nevertheless, vein grafting is still used in many patients and devices to aid vein anastomosis have been introduced by manufacturers. Slit Device belongs to these devices but is not designed to replace the traditional procedures. It is intended only for preventing atheroembolism in a heavily diseased ascending aorta, thus avoiding the need for hypothermic circulatory arrest in patients with unclampable aortas.

## Competing interests

The author has not received any financial support for the construction of the device. For the first two objects the hospital plumber did him a favour. For the other objects the author paid the bill out of his own pocket. Moreover, two invention patents were taken out at the author's own expense.

## Authors' contributions

ET had the idea, drew the object and made the tests.
